# *In silico *identification of NF-kappaB-regulated genes in pancreatic beta-cells

**DOI:** 10.1186/1471-2105-8-55

**Published:** 2007-02-15

**Authors:** Najib Naamane, Jacques van Helden, Decio L Eizirik

**Affiliations:** 1 Laboratory of Experimental Medicine, Université Libre de Bruxelles, Route de Lennik, 808, CP 618, B-1070 Brussels, Belgium; 2 Service de Conformation des Macromolécules Biologiques et de Bioinformatique, Université Libre de Bruxelles, CP 263, Campus Plaine, Blvd du Triomphe, B-1050 Brussels, Belgium

## Abstract

**Background:**

Pancreatic beta-cells are the target of an autoimmune attack in type 1 diabetes mellitus (T1DM). This is mediated in part by cytokines, such as interleukin (IL)-1β and interferon (IFN)-γ. These cytokines modify the expression of hundreds of genes, leading to beta-cell dysfunction and death by apoptosis. Several of these cytokine-induced genes are potentially regulated by the IL-1β-activated transcription factor (TF) nuclear factor (NF)-κB, and previous studies by our group have shown that cytokine-induced NF-κB activation is pro-apoptotic in beta-cells. To identify NF-κB-regulated gene networks in beta-cells we presently used a discriminant analysis-based approach to predict NF-κB responding genes on the basis of putative regulatory elements.

**Results:**

The performance of linear and quadratic discriminant analysis (LDA, QDA) in identifying NF-κB-responding genes was examined on a dataset of 240 positive and negative examples of NF-κB regulation, using stratified cross-validation with an internal leave-one-out cross-validation (LOOCV) loop for automated feature selection and noise reduction. LDA performed slightly better than QDA, achieving 61% sensitivity, 91% specificity and 87% positive predictive value, and allowing the identification of 231, 251 and 580 NF-κB putative target genes in insulin-producing INS-1E cells, primary rat beta-cells and human pancreatic islets, respectively. Predicted NF-κB targets had a significant enrichment in genes regulated by cytokines (IL-1β or IL-1β + IFN-γ) and double stranded RNA (dsRNA), as compared to genes not regulated by these NF-κB-dependent stimuli. We increased the confidence of the predictions by selecting only evolutionary stable genes, i.e. genes with homologs predicted as NF-κB targets in rat, mouse, human and chimpanzee.

**Conclusion:**

The present *in silico *analysis allowed us to identify novel regulatory targets of NF-κB using a supervised classification method based on putative binding motifs. This provides new insights into the gene networks regulating cytokine-induced beta-cell dysfunction and death.

## Background

Pancreatic insulin-producing beta-cells are selectively destroyed by the immune system in type 1 diabetes mellitus (T1DM). The autoimmune assault causes beta-cell dysfunction and death via direct contact with activated immune cells, such as macrophages and lymphocytes, and/or by exposure to soluble mediators secreted by these cells, such as pro-inflammatory cytokines, oxygen free radicals and nitric oxide (NO). The cytokines interleukin (IL)-1β, interferon (IFN)-γ and tumor necrosis factor (TNF)-α induce beta-cell death mainly by apoptosis in rodent and human islets of Langerhans [1]. Beta-cell apoptosis is a complex and highly regulated process that depends on the expression of a large number of pro- and anti-apoptotic genes [2].

Using microarray analyses, we have identified diverse beta-cell gene networks regulated by IL-1β and IFN-γ [3-7]. Cytokines induce stress response genes that are either protective or deleterious for beta-cell survival, whereas genes related to differentiated beta-cell functions are down-regulated. Several of the cytokine effects in beta-cells depend on the activation of the transcription factor (TF) nuclear factor (NF)-κB [2,3]. NF-κB is a homo- or hetero-dimeric complex of proteins from the Rel/NF-κB family, which includes p65, c-rel, relB, p50/p105 and p52/p100. In non-simulated cells NF-κB is located in the cytoplasm as an inactive protein associated with the inhibitor of NF-κB (IκB)α. When cells are simulated by agonists such as cytokines, bacterial products or viruses, IκBα is phosphorylated on serines 32 and 36 by an IκB kinase complex and degraded in the proteasome. This allows NF-κB to translocate to the nucleus where it binds to a set of related DNA target sites (κB-sites) and regulates gene expression [8].

Depending on the cell type and stimulatory cue NF-κB can exert anti- or pro-apoptotic functions [8,9]. Inhibition of cytokine-induced NF-κB activation protects pancreatic beta-cells *in vitro *[10] and *in vivo *[11] against apoptosis, suggesting that NF-κB is mostly pro-apoptotic in beta-cells. To identify cytokine-regulated and NF-κB-dependent beta-cell gene networks, we performed a microarray analysis in cytokine-treated rat beta-cells in which NF-κB activation was blocked by an NF-κB super-repressor (IκB^(SA)2^). By this approach, 66 cytokine-modified and NF-κB regulated genes were identified, including genes coding for cytokines and chemokines and several TFs such as c-Myc, C/EBPβ and C/EBPδ [4]. NF-κB was also found to control, via induction of inducible nitric oxide synthase (iNOS) and NO production, the expression of other TFs such as growth arrest and DNA damage (Gadd)153 and pancreatic duodenal homeobox (PDX)-1. This study was, however, limited to a single time point (24 h), and was based on an array with capacity to detect only ~8,000 probes; thus it did not allow a broad detection of the different genes regulated by NF-κB in beta-cells.

Detailed knowledge of the patterns of gene expression involved in beta-cell death, together with a better understanding on their regulation, is crucial to understand and prevent beta-cell loss in T1DM. Microarray technology allows robust massive gene expression, and we have employed this tool with success for the initial studies on beta-cell gene networks [3-7]. Discovering gene networks, however, requires frequent usage of microarrays at different time points, with and without blockers of specific transcription factors. This demands large amounts of cells, posing a major problem when dealing with rare cells such as primary beta-cells. Moreover, since there is cross talk between different networks, blocking transcription factors is seldom specific. Validation of molecular regulation of beta-cell gene expression has been done by molecular biology techniques such as gel shift assay, transient transfection assay and chromatin immunoprecipitation. These techniques are time consuming (1–2 years of work per gene) and only allow the study of transcriptional regulation of one gene at a time [12-15]. Clearly, novel approaches are required to elucidate the nature of large regulatory systems organized as networks [16].

To obtain comprehensive information on the NF-κB-regulated gene networks in beta-cells, we presently utilized a bioinformatics approach [17] to predict potential NF-κB-responsive genes. An increasing number of studies have used *in silico *analysis of regulatory sequences to assist the laboratory-based approaches in the search of TF targets [18]. Some DNA sequence-based approaches used to decipher regulatory networks relies on the prior knowledge of transcription factor binding site (TFBS) preferences (which can be modelled as a position-specific scoring matrix (PSSM)) [19], whereas others discover new binding sites without prior consideration of the identity of the binding factor [20].

Predicting TFBSs in gene promoter regions using PSSMs is limited by the high number of false matches due to the low information content of the often short and degenerate TFBSs [21]. Consequently, it is necessary to use additional information on gene regulation to improve the correlation between *in silico *predictions and *in vivo *functional binding sites. TFs are often part of cis-regulatory modules (CRMs) [21], and the presence of multiple binding sites for a particular TF in the upstream region of a gene increases the likelihood that the TF truly binds the gene [22]. Moreover, regulatory sequences are often preserved through evolution by selective pressure [23]. Thus, conserved TFBSs between different species are more likely to be functional. Against this background, we incorporated these three biological properties of gene regulation to increase the accuracy of our predictions using discriminant analysis.

Discriminant analysis is a powerful statistical pattern recognition method widely applied for data analysis in biomedical research [24,25]. It has been successfully utilized to identify yeast genes involved in methionine and phosphate metabolism on the basis of upstream regulatory motifs [17]. Discriminant analysis uses a training set to learn how to recognize targets for a given TF based on putative regulatory elements present in their promoter regions. We presently utilized this classification method for the first time in a mammalian system to identify new genes potentially regulated by NF-κB in pancreatic beta-cells. For this purpose, the initial analysis searched a set of 120 known NF-κB target genes (positive examples) for TFBSs over-representation using TFM-Explorer tool [26]. The top matching scores of the most significant over-represented PSSMs in this positive control set were then used to describe the 1 kb upstream sequences from the transcription start site (TSS) of both positive and negative examples of NF-κB regulation (120 genes each). This dataset was then used to train and test two alternative methods for discrimination of NF-κB target genes, namely linear and quadratic discriminant analysis (LDA and QDA). Following these preliminary steps, a large group of human and rat beta-cell genes, detected in our previous array analysis [3-7] was then searched for potential NF-κB target genes. To further increase the reliability of the predictions, we performed a conservation-based filter taking into account the number of homologous upstream regions that are predicted as NF-κB targets in other genomes, namely rat, mouse, human and chimpanzee. Validation of the *in silico *analysis was achieved by comparison with our previous microarray gene expression data obtained from beta-cells exposed to different NF-κB-dependent stimuli.

## Results

### Over-represented TFBSs in the upstream sequences of NF-κB regulated genes

PSSMs are commonly used to model and then to search putative TFBSs in new sequences [21,27,28]. The TFM-Explorer program [26] detects locally over-represented TFBSs, modeled by PSSMs, in a set of coexpressed or coregulated genes. The program first localizes all potential TFBSs for a database of PSSMs, identifies regions where a given PSSM is over-represented and then assesses their statistical significance. We used TFM-Explorer with all available vertebrate PSSMs of the TRANSFAC database [29] (243 matrices) to search for PSSMs with a local over-representation in a set of 120 upstream sequences of known NF-κB target genes, as compared to two sets of 5000 genes randomly picked from the human or mouse genomes (Table 1). As expected, five of the most significant matrices (M00052, M00053, M00054, M00119, M00248) correspond to the main members of the Rel/NF-κB family, whereas the last one (M00117) corresponds to C/EBP-β, which is one of the isoforms of the C/EBP transcription factor.

**Table 1:** Locally over-represented PSSMs in the upstream sequences of 120 known NF-κB-regulated genes

Factor	Matrix ID	P-value (human)	P-value (mouse)
NF-κB	M00052	3.16e-08	2.75e-09
c-Rel	M00053	3.01e-07	2.64e-07
NF-κB	M00054	1.77e-04	1.55e-05
NF-κB	M00208	2.59e-04	1.07e-04
NF-κB	M00194	4.66e-04	3.13e-04
C/EBPβ	M00117	1.43e-02	2.44e-02

Factor: name of the TF; Matrix ID: the TRANSFAC database identifier of the PSSM; P-value: statistical significance of the over-representation of the factor when compared to a background distribution of predicted binding sites in arbitrary human or mouse promoters (Chi square test).

These over-represented matrices were used to scan and characterise the upstream sequences of beta-cell expressed genes by the *patser *program (see Methods). Since the presence of multiple high matrix-scores for a given TF in the upstream region of a gene increases probability of binding, each TFBS was represented by its five top matching scores. Subsequently, each gene upstream sequence was characterized by a 30-element matrix matching score vector. Note that the matrices used for scanning the sequences are partly redundant, since several of them represent the binding specificity of NF-κB. This type of redundancy is however efficiently treated by discriminant analysis.

### Performance of discriminant analysis

**Figure 1:**
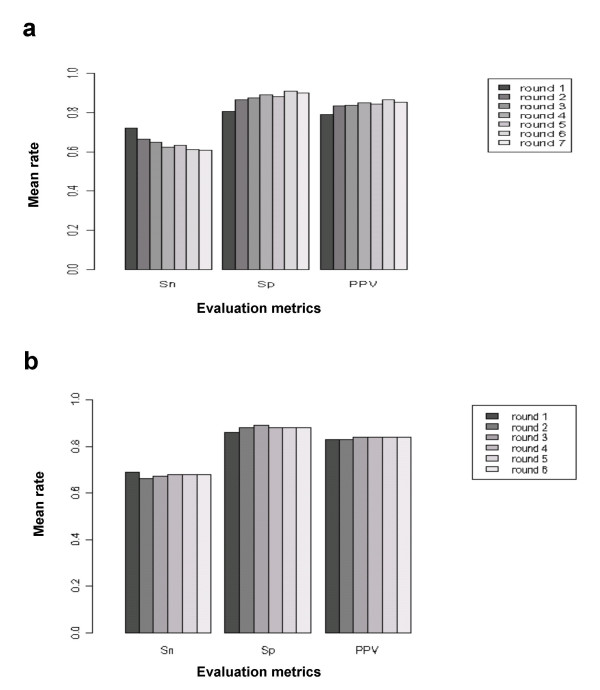
**Average performance of different iterative predictive discriminant analysis approaches**. (**a**) Linear Discriminant Analysis. (**b**) Quadratic Discriminant Analysis. Sensitivity (Sn), Specificity (Sp) and Positive Predictive Value (PPV) are reported for each cycle of the iterative predictive discriminant analysis.

A stratified five-fold cross-validation procedure was performed to estimate the predictive performance of both LDA and QDA. For this purpose, the calibration dataset was first divided into five subsets of equal size (50% each for the positive and negative examples of NF-κB regulation). Each subset was used once for testing the accuracy of the discriminant analysis trained on the four other subsets. LDA and QDA were then applied to each training set using the iterative procedure described in Methods. At each round of the iterative procedure the performance metrics, namely sensitivity, specificity and positive predictive value (PPV) of the classifier on the calibration dataset, were represented in Figure 1 as the average of their observed values in each test set. For both methods the specificity and PPV showed a trend for increase after each round of the iterative procedure while there was a minor decrease in sensitivity, but the LDA performed slightly better than QDA. The best results, in terms of specificity and PPV, were obtained at the 6^th ^round of the iterative procedure for LDA (61% average Sensitivity, 91% average Specificity and 87% average PPV) with an average rate of instances removed from the calibration dataset at around 24.5% (Fig. 1A). For QDA (Fig. 1B) three rounds of the iterative procedure (dataset average reduction of 23.5%) were sufficient to reach the best performance statistics (67% average Sensitivity, 89% average Specificity and 84% average PPV). Since the objective of this study was to predict an experimentally verifiable set of putative NF-κB regulated genes, we selected specificity and PPV as the main criterions of classification. Against this background, the performance level obtained with LDA was considered acceptable, and this method was selected for prediction of regulatory targets of NF-κB in all beta-cell-expressed genes (see below).

### Prediction of NF-κB target genes in beta-cells

**Figure 2:**
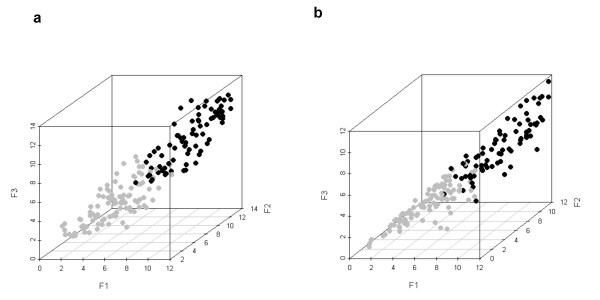
**Separation between NF-κB-regulated (black circles) and non-regulated (gray circles) genes in the pre-processed calibration sample**. (**a**) The axis of the 3D plot represents the first top scores of the matrices M00054 (F1), M00194 (F2), and M00228 (F3) corresponding to the upstream sequences of the calibration sample after an iterative LDA. (**b**) The three top matching scores of the matrix M00054 are represented for each gene. F1: first score; F2: second score; F3: third score.

The whole calibration dataset (120 positive and 120 negative examples) was used to construct a linear classifier for the prediction of NF-κB target genes in beta-cells. An iterative LDA procedure was performed using the original dataset. At the 6^th ^round the resulting pre-processed dataset and the selected variables by the stepwise procedure (see Methods) were retrieved to construct the final LDA classifier. The iterative procedure reduced the number of elements in the original dataset by removing the noisy patterns and returned a pre-processed dataset of 81 NF-κB target genes (dataset reduction of 32.5%) and 99 NF-κB non-target genes (dataset reduction of 17.5%). In Figure 2, the top matching scores obtained from the upstream sequences of this pre-processed dataset were plotted for both three different matrices (Fig. 2A) and an individual matrix (Fig. 2B), allowing visualisation in a three-dimensional space. NF-κB-regulated genes had generally higher scores than the non-regulated ones. The matrix scores-based separation between the two sets of genes was considered acceptable, but there was still a small overlap between the two groups of genes (Figs. 2A and 2B). The LDA classifier trained on this filtered dataset was then used to analyze the genes expressed either in rat primary beta-cells (3575 genes) [3-5], insulin producing INS-1E cells (3068 genes) [6] or in human islets (9443 genes) [7], where we predicted respectively 251, 231 and 580 NF-κB candidate target genes (See Additional files 1 (Table S1), 2 (Table S2) and 3 (Table S3)).

### Phylogeny-based filtering of predicted NF-κB target genes

Groups of genes subject to a specific regulation are assumed to be evolutionary stable. We thus increased confidence in our predictions by filtering putative NF-κB target genes with phylogenetic conservation between two organisms, namely a rodent and a primate. For this purpose the LDA classifier was used to analyse simultaneously the rat, mouse, human and chimpanzee homologous upstream regions of beta-cell-expressed genes. The NF-κB target genes predicted among the genes expressed in primary rat beta-cells and in human pancreatic islets were then compared with those predicted among their respective homologous from other species. The overlaps between the sets of predicted NF-κB target genes in different species were highly significant in all paired comparisons (Table 2). As expected, the significance of these overlaps were more pronounced between closely related species (i.e., p < 1e-300 for the overlap between human and chimpanzee; evolutionary distance of 4–6 million years [30]). The genes which were predicted as NF-κB targets in at least a rodent and a primate species, for which the evolutionary distances are moderate (82–87 million years; [31]), represent 29% (74 genes), 31% (72 genes) and 19% (107 genes) of the total number of predicted NF-κB target genes in primary rat beta-cells, INS-1E cells and human pancreatic islets, respectively (see Additional files 4 (Table S4), 5 (Table S5) and 6 (Table S6)).

**Table 2:** Comparison of predicted NF-κB-regulated genes in different species

Species 1	Species 2	Targets in 1	Targets in 2	Overlap	Total # of genes	P-value
Rat	mouse	217	230	82	2688	3.7e-37
Rat	chimpanzee	205	281	63	2540	6.2e-16
Rat	human	218	310	64	2666	6.9e-14
Human	rat	504	296	66	7545	3.7e-19
Human	mouse	541	355	81	7915	2.1e-23
Human	chimpanzee	536	470	353	8196	<1e-300

Targets in 1: number of NF-κB targets in species 1; Targets in 2: number of NF-κB targets in species 2; Overlap: number of NF-κB targets in common between species 1 and 2; Total # of genes: number of analyzed pairs of homologous genes; P-value: probability to observe at least the obtained common number of NF-κB targets (calculated on the basis of the hypergeometric distribution).

### Comparison of *in silico *analysis against microarray data

**Figure 3:**
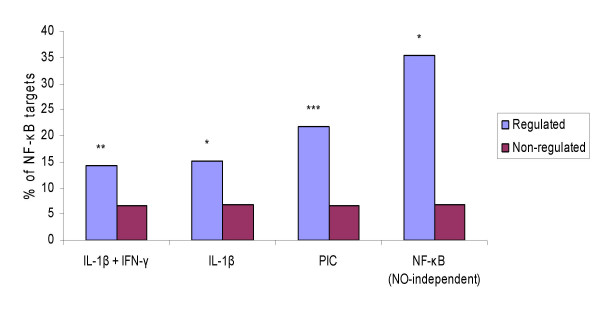
**Enrichment of predicted NF-κB target genes in genes regulated by putative NF-κB-dependent stimuli**. The statistical significance of the abundance of predicted NF-κB target genes is assessed in sets of genes regulated by cytokines (IL-1β alone or IL-1β +IFN-γ) or double stranded RNA (tested in the form of polyinosinic-polycytidylic acid, PIC) against sets of genes which do no respond to these stimuli. The set labeled as "NO-independent" indicates that NO-dependent genes were removed, since they may be indirectly regulated by NO production. *P < 0.005; **P < 0.0005; ***P < 0.0001 *vs*. non-regulated gene set (Fisher's exact test). Microarray data was obtained from [3-6].

To further assess the validity of the *in silico *target predictions, the results described above were compared with those of previous microarray studies from our group (Fig. 3). The microarray datasets describe the transcriptional response of beta-cells to putative NF-κB-dependent stimuli such as cytokines [3], cytokines in the presence of an NF-κB blocker [4] or an iNOS blocker [6] and double stranded RNA (dsRNA) [5].

When comparing the present *in silico *findings against mRNAs that are up- or down-regulated by 6 or 24 h exposure of primary purified rat beta-cells to IL-1β and/or IFN-γ [3], we found that 27 of 190 cytokine (IL-1β + IFN-γ)-simulated genes (14.2%) and 15 of 99 IL-1β-regulated genes (15.2%) were predicted as NF-κB target genes in rat beta-cells (see Additional file 7: Table S7). In both cases there was a nearly 2-fold enrichment for putative NF-κB target genes as compared to non-cytokine regulated genes (p < 0.005; Fisher's exact test). We also checked the enrichment of predicted NF-κB target genes in a list of 84 dsRNA-simulated genes [5]. 19 of the genes in this list (21%) (see Additional file 7: Table S7) and only 234 of 3488 non-dsRNA-induced genes (6.7%) were predicted as NF-κB target genes, with a 3-fold enrichment (p < 10^-5^; Fisher's exact test).

A combination of NF-κB blocking with microarray analysis [4], has identified 66 cytokine-induced and NF-κB-regulated (direct or indirect targets) genes in primary rat beta-cells. Of note, this study used only one late time point, namely 24 h. 53 of the 66 genes were present in the set of beta-cell-expressed genes. To render the comparison more reliable, NO-regulated genes were removed from this list. It has been shown by a time course microarray analysis [6] that cytokines induce a late NO production which indirectly modifies the expression of nearly 50% of the cytokine-affected mRNAs after 12 h. Among the 53 NF-κB-regulated genes, 17 are NO-independent (putative direct targets) [6] and 6 of these cytokine induced, NF-κB-regulated and NO-independent genes (see Additional file 7: Table S7) were predicted as putative NF-κB target genes by the LDA classifier (35.3%, p < 0.0007; Fisher's exact test).

### Functional classes and temporal gene expression clusters enriched in predicted NF-κB target genes

TFs often regulate groups of genes with similar expression profiles and/or related function [32]. To test if this was the case in putative NF-κB-dependent genes, 225 NO-independent genes were retrieved from nearly 500 cytokine-regulated genes. These genes were previously classified into 14 different groups according to their putative function and 15 clusters according to their temporal expression profile [6]. These datasets were tested for significant differences in the distribution of the functional classes between predicted NF-κB target and non-target genes (Table 3). There were 32 NF-κB target genes and 193 NF-κB independent genes among the 225 NO-independent and cytokine-regulated genes. The NF-κB-dependent genes are listed in Table 4. The most significantly over-represented functional classes in the set of predicted NF-κB target genes were cytokines and chemokines, major histocompatibility complex (MHC)-related genes and adhesion molecules. Concerning the temporal clusters, "cluster 1" (see [6]) was the only one with a significant over-representation in the group of NF-κB target genes. This cluster is characterized by an early peak of gene expression at 2 h, followed by a decrease to below control levels at 6 h, and then a return to basal expression at 24 h. 38% of the genes in this cluster are related to signal transduction, 17.4% are transcriptions factors and only 26% are NO-dependent.

**Table 3:** Distribution of functional classes and temporal gene expression clusters between predicted NF-κB target and non-target genes in INS-1 cells.

Temporal cluster	P-value
cluster 1	0.006
cluster 2	1
cluster 3	0.66
cluster 4	1
cluster 5	0.36
cluster 6	0.32
cluster 7	0.87
cluster 8	0.73
cluster 9	1
cluster 10	0.14
cluster 11	0.64
cluster 12	0.66
cluster 13	0.87
cluster 14	0.53
cluster 15	1

Functional class	P-value

Metabolism	0.65
Protein synthesis	0.87
Ionic channels	0.48
Hormones and growth factors	1
Cytokines, chemokines	0.05
Signal transduction	0.94
MHC related	0.06
Cell adhesion	0.03
Transcription factors	0.45
RNA synthesis	1
Cell cycle	1
Defense repair	0.79
Apoptosis ER stress	1
Miscellaneous	0.81

The classifications involved 225 NO-independent and cytokine-regulated genes in INS-1E cells [6]. 32 of them were predicted as NF-κB targets by the present approach. P-value: statistical significance of the overlap between the genes belonging to the functional or temporal class and the set of predicted NF-κB target genes (hypergeometric probability distribution).

**Table 4:** List of cytokine-regulated and NO-independent genes predicted as NF-κB targets in primary rat beta-cells.

**Ensembl gene ID**	**Gene description**	**Prob.**
ENSRNOG00000011023	Nitric oxide synthase, inducible	0.999999
ENSRNOG00000022256	Small inducible cytokine B10 precursor	0.9999876
ENSRNOG00000014297	Syndecan-4 precursor	0.9998138
ENSRNOG00000002792	Macrophage inflammatory protein 2 precursor	0.9995781
ENSRNOG00000007390	NF-κB inhibitor alpha	0.9992648
ENSRNOG00000000105	Complexin-2	0.9985486
ENSRNOG00000018735	H-2 class II histocompatibility antigen, gamma chain	0.9977451
ENSRNOG00000018273	Nucleolin	0.9966934
ENSRNOG00000030712	RT1 class Ia, locus A1	0.9964791
ENSRNOG00000000451	RT1 class II, locus Ba	0.9949906
ENSRNOG00000016346	Protein kinase C, delta type	0.9937999
ENSRNOG00000002802	Growth regulated alpha protein precursor	0.9917401
ENSRNOG00000021128	ATP-sensitive inward rectifier potassium channel 11	0.9899182
ENSRNOG00000031607	RT1 class I, CE4	0.9867331
ENSRNOG00000014288	Fibronectin precursor	0.9827045
ENSRNOG00000009980	Lipid phosphate phosphohydrolase 1	0.977648
ENSRNOG00000022719	Multidrug resistance protein 1	0.9542411
ENSRNOG00000019048	Superoxide dismutase 2, mitochondrial	0.946606
ENSRNOG00000000837	Tumor necrosis factor precursor	0.9223781
ENSRNOG00000018524	Ezrin	0.9157411
ENSRNOG00000001989	CD166 antigen precursor	0.9117247
ENSRNOG00000000763	RT1 class Ib gene RT1-M3	0.8871843
ENSRNOG00000006877	Ephrin-B1 precursor	0.8616659
ENSRNOG00000017496	2',3'-cyclic-nucleotide 3'-phosphodiesterase	0.8035472
ENSRNOG00000003897	Collagen alpha-1	0.73606
ENSRNOG00000012410	S-100 protein, alpha chain.	0.7144861
ENSRNOG00000008144	Interferon regulatory factor 1	0.6926852
ENSRNOG00000006375	Voltage-dependent anion-selective channel protein 1	0.604003
ENSRNOG00000007237	DNA-binding protein inhibitor ID-2	0.5668806
ENSRNOG00000015787	Synaptonemal complex protein SC65.	0.5488754
ENSRNOG00000010708	Transcription factor GATA-4	0.5444669
ENSRNOG00000016552	Hydroxymethylglutaryl-CoA synthase, cytoplasmic	0.5286608

Prob.: Posterior probability for NF-κB regulation.

**Figure 4:**
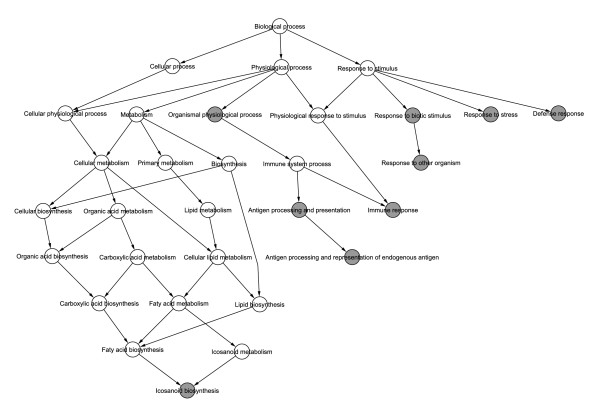
**Hierarchical view of the Gene Ontology (GO) analysis results for predicted NF-κB target genes**. The gray circles are statistically over-represented GO biological processes within the predicted NF-κB target genes as compared to the NF-κB non target genes (FDR adjusted *p*-value < 0.05 from Fisher's exact test). The Biological process node is the root of the GO biological processes. Downstream terms in the GO hierarchy provide more specific annotations. Additional information, including lists of all biological processes assigned to the analysed genes, is provided in Additional files 8, 9 and 10.

The NF-κB candidate target genes were also searched for statistical associations with the annotations from Gene Ontology (GO). Relevant GO biological processes were extracted from the three sets of NF-κB candidate target genes in rat primary beta-cells (251 genes), insulin producing INS-1E cells (231 genes) or in human pancreatic islets (580 genes). The GO analysis (Figure 4; for statistical details see Additional files 8 (Table S8), 9 (Table S9) and 10 (Table S10)) indicated significant over-representation of biological processes such as "immune response", "antigen presentation and processing", "response to biotic stimulus" and "defense response".

## Discussion

Identification of TF target genes by computational approaches poses more difficulties in higher eukaryotes than in organisms with smaller genomes such as yeast. Scanning of large mammalian genomes for PSSMs matches is done in an enormous sequence space, as compared to the short size of DNA motifs recognized by TFs [33]. This leads to a poor accuracy in TF target prediction, since only a small fraction of the predicted binding sites will have a functional role [21]. To reduce false predictions, the present study was restricted to the 1 kb upstream of the TSS for each gene. In line with other studies [34,35], we have previously observed that most PSSM-predicted NF-κB binding sites are located within 1 kb upstream the TSS [36]. This restriction, however, may decrease sensitivity and contribute for the fact that some known target genes escaped detection.

PSSM-based approaches for TFBSs prediction often relay on broad and sometimes inaccurate assumptions, and do not take in consideration putative combinatorial interactions between TFs that recognize multiple sites [21]. To incorporate such biological annotation to the prediction of NF-κB target genes, we first searched for common regulatory elements in the upstream sequences of a set of known NF-κB target genes. In addition to the PSSMs of the different members of the Rel/NF-κB family, one PSSM corresponding to C/EBP was also over-represented in the set of positive controls (Table 1). NF-κB and C/EBP are known to interact, and their binding sites combine and form regulatory modules for several genes [37,38]. Moreover, matrix-based methods have been already used to predict genes with composite NF-κB:C/EBP regulatory sites [39]. To account for the frequent presence of multiple binding sites for the same TF in a given regulatory region, we detected multiple hits from each locally over-represented PSSM to characterize individual upstream sequences, and used them as input to train the classifier.

Alignment-based phylogenetic footprinting methods are widely used to improve the specificity of TF target genes prediction [28,40]. These methods, however, rely on the assumption that the regulatory regions are sufficiently conserved to be aligned. Instead of searching for putative TFBS that are situated in conserved regions in alignment between orthologous sequences, we used our classifier to screen each gene in parallel with a set of its homologs in other species: a given gene was considered as an NF-κB target only if its homologs were also classified as NF-κB regulated genes. This improved the accuracy of our predictions (several of the genes identified by this approach have been previously shown in other tissues to be NF-κB-dependent [41,42]), but lead to an 70–80% decrease in the number of hits. In other words, although this approach apparently decreases false positives, it leads to a large increase in false negatives. Thus, manganese superoxide dismutase (MnSOD) and c-Myc, known NF-κB dependent genes [14,43], were lost following this step.

The validation of computational methods is crucial to assess the significance of bioinformatics predictions. One of the possible ways for validation is the use of global gene expression intersection [44]. Thus, we utilized microarray datasets from our group reporting the transcriptional response of beta-cells to different putative NF-κB-dependent stimuli [3-6]. By comparing them to our *in silico *predictions we observed that, in general, genes regulated by putative NF-κB-dependent stimuli had a 2–3-fold higher probability to be predicted as NF-κB targets than non-responsive genes (p < 0.005). Considering that these microarray experiments included few time points and a limited set of genes, and that gene expression can also be regulated by variables such as chromatin configuration, which is not detected by *in silico *approaches, the 20–30% agreement between our predictions and actual gene expression data is reasonable. Functional classes such as cytokines and chemokines, MHC-related genes and adhesion molecules (Table 3), whose expression is known to be regulated by NF-κB in other tissues [45,46], were significantly enriched in a set of 32 manually annotated genes predicted as NF-κB targets. In agreement with these observations, GO analysis indicated that categories such as "immune response" and "antigen presentation and processing" are over-represented in putative NF-κB-dependent genes. In addition, the temporal expression profile described by the enriched temporal "cluster 1" (see [6]) is consistent with NF-κB regulation.

The predictions obtained from the present analysis represent manageable gene lists for further experimental validation, and provide an integrated platform for deciphering the NF-κB dependent gene networks in beta-cells. Several genes in these lists are already described as NF-κB dependent in beta-cells and/or in other tissues, suggesting that the discrimination procedure generated reliable results. For instance, the cytokine-induced gene iNOS, for which the role of NF-κB was already confirmed in beta-cells [13], was predicted with the highest posterior probability in primary rat beta-cells (Table 5). Among the genes already described as NF-κB targets in other tissues, we predicted with high probability some cytokines and chemokines such as the macrophage inflammatory protein 2 (MIP-2) [47] and the small inducible cytokines B10 (IP-10) [48]. Most of the remaining genes, however, are novel potential NF-κB targets, and those among them which are predicted with the highest probabilities in different organisms can be interesting candidates for detailed experimental analysis.

**Table 5:** List of primary rat beta-cell genes predicted as NF-κB targets in both rat and a primate species (top 30 genes).

**Ensembl gene ID**	**Gene description**	**Prob.**	**Human**	**Mouse**	**Chimpanzee**
ENSRNOG00000011023	**Nitric oxide synthase, inducible**	0.999999	NF-κB	NF-κB	NF-κB
ENSRNOG00000022256	**Small inducible cytokine B10 precursor**	0.999988	NF-κB	NF-κB	NF-κB
ENSRNOG00000014297	**Syndecan-4 precursor**	0.999814	NF-κB	NF-κB	NF-κB
ENSRNOG00000012180	RAB5A, member RAS oncogene family	0.999691	NF-κB	NF-κB	NF-κB
ENSRNOG00000020102	sirtuin	0.999613	NF-κB	NF-κB	NF-κB
ENSRNOG00000002792	**Macrophage inflammatory protein 2 precursor**	0.999578	NF-κB	NF-κB	NF-κB
ENSRNOG00000021130	Sulfonylurea receptor 1	0.999543	NF-κB	NF-κB	CTL
ENSRNOG00000021156	PREDICTED: vascular endothelial growth factor B	0.999392	NF-κB	NF-κB	NF-κB
ENSRNOG00000007390	**NF-κB inhibitor alpha**	0.999265	NF-κB	NF-κB	NF-κB
ENSRNOG00000013412	cAMP response element binding protein	0.999217	NF-κB	NF-κB	NF-κB
ENSRNOG00000002843	**Small inducible cytokine B5 precursor**	0.999081	NF-κB	NF-κB	NF-κB
ENSRNOG00000008217	**Mucosal addressing cell adhesion molecule 1 precursor**	0.998906	NF-κB	NF-κB	NA
ENSRNOG00000020481	Platelet-activating factor acetylhydrolase IB gamma subunit	0.998769	NF-κB	NF-κB	NF-κB
ENSRNOG00000031090	RT1 class I, CE7	0.99778	NF-κB	NF-κB	NF-κB
ENSRNOG00000000723	RT1 class I, CE5 isoform 2	0.99716	NF-κB	NF-κB	NF-κB
ENSRNOG00000011559	Calponin-3	0.996972	NF-κB	NF-κB	NF-κB
ENSRNOG00000000468	RT1 class I, A3	0.996707	NF-κB	CTL	NF-κB
ENSRNOG00000030251	RT1-CE10 protein	0.996647	NF-κB	NF-κB	NF-κB
ENSRNOG00000030712	RT1 class Ia, locus A1	0.996479	NF-κB	NF-κB	NF-κB
ENSRNOG00000005438	Neuroendocrine convertase 2 precursor	0.995956	NF-κB	NF-κB	NF-κB
ENSRNOG00000032707	Pro-epidermal growth factor precursor	0.993999	NF-κB	NF-κB	NF-κB
ENSRNOG00000016346	Protein kinase C, delta type	0.9938	NF-κB	NF-κB	CTL
ENSRNOG00000006268	**Interferon beta precursor**	0.99279	NF-κB	NF-κB	NF-κB
ENSRNOG00000016630	Tln_predicted protein.	0.989963	NF-κB	CTL	NF-κB
ENSRNOG00000019080	hydroxy-delta-5-steroid dehydrogenase, 3 beta- and steroid delta-isomerase 7	0.987715	NF-κB	NF-κB	NF-κB
ENSRNOG00000031607	RT1 class I, CE4	0.986733	NF-κB	CTL	NF-κB
ENSRNOG00000004148	Serine/threonine-protein kinase PCTAIRE-2	0.98582	CTL	NF-κB	NF-κB
ENSRNOG00000028387	START domain containing 3	0.985143	NF-κB	CTL	NA
ENSRNOG00000003743	Aspartyl-tRNA synthetase	0.984986	NF-κB	CTL	CTL
ENSRNOG00000016588	myeloid-associated differentiation marker	0.983779	NF-κB	NA	NF-κB

Known NF-κB target genes are in bold; Prob.: Posterior probability for NF-κB regulation; NF-κB: the homolog gene is predicted as an NF-κB target; CTL: the homolog gene is predicted as a non NF-κB target; NA: upstream sequence of the homolog gene not available.

Improvement of *in silico *analysis may be achieved by a more efficient integration of other types of genomic data. For instance by adding gene expression profiles to the matrix score vectors describing the upstream sequences, we can provide an important discriminative criterion to the classifier. Expression profiles of genes regulated by the same TF are often highly correlated [32], and addition of this information to the classifier may improve prediction specificity.

## Conclusion

The sequencing of the human, rat and mouse genomes [34,49-51] allows the development of new approaches to determine global cellular regulatory mechanisms by *in silico *sequence analysis. In the present work discriminant analysis has been successfully applied to identify novel NF-κB-regulated genes in pancreatic beta-cells. The discriminant classifier was developed based on the matrix score profiles of putative TFBSs in the upstream sequences of NF-κB-regulated and non-regulated genes and showed reasonable predictive power. The results obtained provide new insights into the modeling of gene networks regulating cytokine-induced beta-cell dysfunction and death, and open several new avenues for research. In future work, the method will be improved and applied for detecting the regulatory targets of other TFs, such as STAT-1, that also regulate key beta-cell genes implicated in beta-cell death [52].

Microarray gene expression data, in combination with the present and future *in silico *sequence analysis, will hopefully provide valuable tools to unravel the architecture of key beta-cell gene networks. The *in silico *work will help to characterize gene clusters regulated by similar transcription factors, and will focus the laborious promoter studies on selected genes. This combined approach will identify the genetic network structure of beta-cells and might generate new targets for drug design and imaging. For instance, and based on this approach, we have already developed *in vivo *approaches to prevent experimental diabetes by blocking NF-κB [11] and STAT-1 [52] and identified several interesting targets for beta-cell imaging (Flamez D, Kutlu B, Goodman N and Eizirik DL, unpublished data).

In conclusion, the present approach constitutes a "proof of principle" for the integrated use of functional genomics [3-7] and bioinformatics ([36]; present study) in the detailed molecular characterization of a relevant cell type for human pathology. By following this integrated approach, we expect to fully map the interacting networks of genes and proteins downstream of the pro-apoptotic signals leading to beta-cell death in T1DM. This will allow us to move the search for a cure for T1DM from an empiric and often blind approach to one that is really mechanistically driven – the ultimate outcome being the development of logical and targeted therapies to prevent the disease.

## Methods

### Calibration sets

To train and evaluate the discriminant analysis methods used in this study, we acquired a calibration dataset consisting of putative promoter sequences for positive and negative examples of NF-κB regulation. The set of positive examples was extracted from a compilation of genes known to contain functional NF-κB binding sites from diverse tissues of human, mouse and rat [46]. From this collection we selected 96 human, 17 mouse and 7 rat genes with a strong experimental evidence for NF-κB binding. As negative examples we selected 120 genes with the least significant changes in expression in a microarray analysis where rat beta-cells were stimulated by cytokines [3]. These genes are supposed not to be regulated by NF-κB.

### Upstream sequence collections

We analyzed the promoters of sets of genes expressed in rat primary beta-cells (3575 genes) [3-5], insulin-producing INS-1E cells (3068 genes) [6] or human islets (9443 genes) [7]. For each gene the 1 kb upstream sequence, starting from the TSS, was retrieved from the ENSEMBL database (release 35, [53]) and analyzed as explained in the next section. The choice of the 1 kb limit for upstream sequence was based on findings on rodent genomes indicating that most annotated TFBSs are located at this position [34].

### PSSM selection and binding site scoring

The web application TFM-Explorer [26] was used to determine PSSMs enriched in NF-κB target genes. This program identifies all potential TFBSs in the set of promoter sequences using all available vertebrate matrices of the matrix library collected in the TRANSFAC database. It reports statistically significant regions where predicted binding sites show local over-representation. The top six significant matrices discovered by this method were used to scan both the upstream sequences present in the calibration dataset and those corresponding to genes expressed in primary rat beta-cells, INS-1E cells and human pancreatic islets. This scanning step was performed using the pattern-matching program *patser *[54]. For a given PSSM of width *w*, the *patser *program slided a window of length *w *along both strand sequences and assigned a score to each position; the top five matching scores were retrieved for each analyzed upstream sequence. Each upstream sequence was thus represented by a 30-element TFBS matrix score vector (5 top scores × 6 matrices).

### Discriminant methods

Discriminant analysis seeks to find a rule for accurately predicting a categorical response (i.e. regulated *vs*. not regulated) based on a set of measured variables (i.e. TFBS matrix-scores) [24]. Our selected dataset was used to train two discrimination methods, LDA and QDA, for recognizing NF-κB target genes according to the observed matrix matching scores in their promoter regions. The ultimate goal was to allocate a gene to a regulation group, NF-κB target or non target genes, based on the 30-element vector of TFBS matrix scores. In addition to assigning each element to a group (regulated or not), discriminant analysis estimates *posterior probabilities*, indicating the probability for this element to belong to the respective groups and classifying the gene as belonging to the group with the highest posterior probability. Discriminant analysis also allows specifying *prior probabilities *to estimate the fraction of elements expected in the different groups. LDA and QDA differ in that LDA is based on the assumption that the variables are multivariate normally distributed in each group, with different mean vectors but identical covariance matrices, whereas QDA is based on the assumption of group-specific covariance matrices.

### Cross validation, variable selection and noise reduction

#### Stratified 5-fold cross-validation

To evaluate the accuracy of the classification methods utilized in this work, the above described dataset was first divided into five subsets of equal size, with the positive and negative examples of NF-κB regulation represented by the same number of genes. In each experiment four subsets were used for training and the remaining one for testing. Performance statistics were then averaged over the five test folds. Three different statistics were used to evaluate the predictive performance of LDA and QDA. Sensitivity is Sn = TP/(TP+FN), specificity is Sp = TN/(TN+FP) and positive predictive value is PPV = TP/(TP+FP), where TP, TN, FP and FN refer to the number of True Positives, True Negatives, False Positives and False Negatives, respectively.

#### Variable selection

Variable selection is a crucial step in machine learning. Due to the problem of over-fitting many classification methods perform poorly when taking into consideration large numbers of variables [55]. The problem of over-dimensionality is particularly sensible in QDA, since this method considers each pairwise combination of variables. To reduce the number of variables we presently applied a forward stepwise procedure, which starts from an empty set of variables and adds at each step a single variable which produces the greatest improvement in the performance of the classifier [55]. Within each training phase, the forward stepwise variable selection procedure was performed using an internal leave-one-out cross-validation (LOOCV) [55,56]. LOOCV is the extreme case of the k-fold cross validation procedure: if one has N data examples, N experiments will be performed with N-1 training cases and 1 test case. The performance statistics are then averaged over the N test folds.

#### Noise reduction by iterative procedure

Given the protocol for building the calibration dataset, the training groups may themselves contain errors. In particular, the negative control set might contain genes which would be regulated by NF-κB under different conditions than those presently tested. In addition, the positive training set might contain genes for which the binding sites are outside the 1 Kb sequence considered in this analysis. Such erroneous training examples should be removed as they can affect the performance of the discriminant analysis [57]. To address this issue, an iterative procedure was performed within each training phase to reduce noise. The classifier was first trained using the original training subset; then, in the next round of the procedure, the elements which were misclassified during the internal LOOCV were removed, and the remaining examples were used as a pre-processed training subset. The procedure was iterated until the training subset was not any more modified.

### Phylogeny-based filtering of predicted NF-κB target genes

Rat, human, mouse and chimpanzee homologs from ENSEMBL database were retrieved for each gene expressed in rat primary beta-cells, insulin producing INS-1E cells or human pancreatic islets. The discriminant procedure took as input these sets of homologs, predicted NF-κB regulation and returned a posterior probability for each gene.

The classification was initially performed for each organism separately. Then, the overlap predictions (number of genes predicted in both organisms) was computed for each pair of organisms and tested for significance by the hypergeometric distribution using the *compare-classes *program from the Regulatory Sequence Analysis Tools (RSAT) suite [58]. The program *compare-classes *compares two classifications (clustering results, functional classes, etc.) and assesses the statistical significance of common members between each pair of classes by calculating the hypergeometric probability. For the final prediction, we required homologs predicted as NF-κB-regulated genes to be present in at least one rodent and one primate species.

### Comparison of *in silico *analysis against microarray data

The microarray data utilized for the comparison against the present in silico analysis were obtained using the rat genome Affymetrix U34-A Gene Chips containing ~8,000 probes or the human genome U133-A arrays containing 22,000 probe sets corresponding to 14500 distinct genes. Using these arrays, we detected 3575, 3068 and 9443 genes expressed in respectively primary rat beta-cells [3-5], insulin producing rat INS-1E cells [6] and primary human islets [7]. Integrated information on these genes is available at the "Beta-Cell Gene Expression Bank" [59].

To assess the statistical significance of the over-representation of predicted NF-κB-regulated genes we used the Fisher exact probability test for 2 × 2 contingency tables implemented in the statistical package R [60].

From nearly 500 genes described as cytokine-regulated [6], 225 NO-independent genes were retrieved and mapped to the list of 3068 genes expressed in INS-1E cells, resulting in 32 genes predicted as NF-κB-target. The program *compare-classes *was used to detect significant overlaps between annotated classes (14 different functional classes and 15 temporal gene expression clusters, described in [6]), and the subsets of genes predicted as NF-κB targets (32 genes) or not (193 genes).

The FatiGO (Fast Assignment and Transference of Information using Gene Ontology (GO)) web tool [61], available at [62], was used to search for significant differences in distributions of GO:Biological Process (GO:BP) categories between predicted NF-κB-regulated and non-regulated groups of genes. GO:BP categories that were statistically over- or under-represented in the predicted sets of NF-κB target genes were identified by a Fisher's exact test (adjusted p < 0.05) that consider multiple testing. Adjusted *p*-values returned by FatiGO were calculated using the false discovery rate (FDR) [63].

## Authors' contributions

NN was responsible for performing the *in silico *and statistical analysis and comparisons between *in silico *and array data, and for writing the first draft of the manuscript. JvH supervised the *in silico *and statistical analysis and made improvements and suggestions to the manuscript. DLE initiated the study and supervised the biological analysis and the comparisons between *in silico *and array data; he contributed for writing the manuscript and preparing the final draft of the article. All authors have read and approved the final manuscript.

## Supplementary Material

Additional file 1 table S1. list of predicted NF-κB-responding genes in primary rat beta-cells. Prob.: posterior probability for NF-κB regulation; EST: expressed sequence tag. Click here for file

Additional file 2 table S2. list of predicted NF-κB-responding genes in INS1-E cells. Prob.: posterior probability for NF-κB regulation; EST: expressed sequence tag. Click here for file

Additional file 3 table S3. list of predicted NF-κB-responding genes in human islets. Prob.: Posterior probability for NF-κB regulation; NA: gene description not available. Click here for file

Additional file 4 table S4. list of primary rat beta-cell genes predicted as NF-κB targets in both rat and a primate species. Prob.: Posterior probability for NF-κB regulation; NF-κB: the homolog gene is predicted as an NF-κB target; CTL: the homolog gene is predicted as a non NF-κB target; NA: upstream sequence of the homolog gene not available. Click here for file

Additional file 5 table S5. list of INS-1E cell genes predicted as NF-κB targets in both rat and a primate species. Prob.: Posterior probability for NF-κB regulation; NF-κB: the homolog gene is predicted as an NF-κB target; CTL: the homolog gene is predicted as a non NF-κB target; NA: upstream sequence of the homolog gene not available. Click here for file

Additional file 6 table S6. list of human islet genes predicted as NF-κB targets in both human and a rodent species. Prob.: Posterior probability for NF-κB regulation; NF-κB: the homolog gene is predicted as an NF-κB target; CTL: the homolog gene is predicted as a non NF-κB target; NA: upstream sequence of the homolog gene not available. Click here for file

Additional file 7 table S7. list of IL-1β-, cytokine (IL-1β + IFN-γ)- or PIC-regulated primary rat beta-cell genes predicted as NF-κB target genes. Prob.: Posterior probability for NF-κB regulation; IL: gene regulated by IL-1βalone; IFN: gene regulated by IFN-γ alone; IL+IFN: gene regulated by IL-1β + IFN-γ; PIC: gene regulated by dsRNA; NF-κB: gene regulated by NF-κB in microarray analysis; NO: "INDEP" indicates that the gene is NO independent and "DEP" indicates the gene is NO dependent. Click here for file

Additional file 8 table S8. list of all 1802 biological processes assigned to the genes expressed in INS-1E cells. Level: GO level; Target genes: Ensembl identifiers of the NF-κB target genes assigned to the biological process; N° target genes: number of the NF-κB target genes assigned to the biological process; Percentage target genes: percentage of the NF-κB target genes assigned to the biological process; Non target genes: Ensembl identifiers of the NF-κB non target genes assigned to the biological process; N° non target genes: number of the NF-κB non target genes assigned to the biological process; percentage non target genes: percentage of the NF-κB non target genes assigned to the biological process; Unadj. pvalue: p-value from Fisher's exact test without adjusting for multiple comparisons; Adj. pvalue: FDR adjusted *p*-value. Click here for file

Additional file 9 table S9. list of all 1853 biological processes assigned to the genes expressed in primary rat beta-cells. Level: GO level; Target genes: Ensembl identifiers of the NF-κB target genes assigned to the biological process; N° target genes: number of the NF-κB target genes assigned to the biological process; Percentage target genes: percentage of the NF-κB target genes assigned to the biological process; Non target genes: Ensembl identifiers of the NF-κB non target genes assigned to the biological process; N° non target genes: number of the NF-κB non target genes assigned to the biological process; percentage non target genes: percentage of the NF-κB non target genes assigned to the biological process;Unadj. pvalue: p-value from Fisher's exact test without adjusting for multiple comparisons; Adj. pvalue: FDR adjusted *p*-value. Click here for file

Additional file 10 table S10. list of all 2903 biological processes assigned to the genes expressed in human pancreatic islets. Level: GO level; Target genes: Ensembl identifiers of the NF-κB target genes assigned to the biological process; N° target genes: number of the NF-κB target genes assigned to the biological process; Percentage target genes: percentage of the NF-κB target genes assigned to the biological process; Non target genes: Ensembl identifiers of the NF-κB non target genes assigned to the biological process; N° non target genes: number of the NF-κB non target genes assigned to the biological process; percentage non target genes: percentage of the NF-κB non target genes assigned to the biological process;Unadj. pvalue: p-value from Fisher's exact test without adjusting for multiple comparisons; Adj. pvalue: FDR adjusted *p*-value. Click here for file
